# A comprehensive integration of factors affecting vitamin B_12_ concentration in milk of Holstein cows: Genetic variability, milk productivity, animal characteristics, and feeding management

**DOI:** 10.3168/jdsc.2023-0535

**Published:** 2024-05-10

**Authors:** Mélissa Duplessis, Christiane L. Girard, Doris Pellerin, Liliana Fadul-Pacheco, Roger I. Cue

**Affiliations:** 1 Agriculture et Agroalimentaire Canada, Centre de recherche et développement de Sherbrooke, Sherbrooke, J1M 0C8, QC, Canada; 2 Département des sciences animales, Université Laval, Québec, G1V 0A6, QC, Canada; 3 Lactanet, Sainte-Anne-de-Bellevue, H9X 3V9, QC, Canada; 4 Department of Animal Science, McGill University, Sainte-Anne-de-Bellevue, H9X 3V9, QC, Canada

## Abstract

Daily vitamin B_12_ (VB_12_) requirements of humans can naturally be fulfilled by animal product consumption, especially products from ruminants because of bacteria dwelling in their rumen. Indeed, only bacteria can synthesize this vitamin. Milk is hence an excellent source of VB_12_. This cross-sectional study was undertaken to unravel factors, such as genetic variation, diet and cow characteristics, and milk production, explaining the large variation in milk VB_12_ concentration among cows by using an integrative approach. Milk samples from 2 consecutive milkings were collected from 3,533 Canadian Holstein cows (1,239 first, 932 second, and 1,362 third and more lactations) located in 99 herds with various feeding management. For the purpose of genetic variation analysis, pedigrees were traced back for 3 complete generations for each sire and dam. A total of 10,021 identities were used in the subsequent genetic analyses. Milk VB_12_ averaged 4.2 ng/mL with a range between 0.7 and 9.0 ng/mL. Dietary fiber (NDF from forage, dietary NDF, ADF, and lignin) increased and dietary components related to energy (NFC, starch, NE_L_, and percentage of concentrate) decreased VB_12_ in milk. Milk VB_12_ varied with DIM, with a similar pattern as milk fat and protein concentration lactation curves. Milk VB_12_ increased as age at calving increased. When disregarding the herd variance, the heritability value was 0.37, meaning that milk VB_12_ can be modified by genetic selection. The final model including factors related to the diet, animal characteristics and milk productivity, and genetic variation explained 79% (pseudo-R^2^) of the milk VB_12_ variation. When excluding the random effect of the cow (i.e., excluding the animal and genetic relationships), the pseudo-R^2^ dropped to 43%, reinforcing the importance of genetic variation in explaining milk VB_12_ variation. To our knowledge, the present study is the most comprehensive evaluation of factors affecting milk VB_12_ variation including the greatest number of cows from various lactation stages.

## Highlights

• Milk vitamin B_12_ concentration varied from 0.68 to 8.96 ng/mL among cows.
• Heritability was 0.37; hence, milk vitamin B_12_ can be modified by genetic selection.
• The model explained 79% of the milk vitamin B_12_ concentration variation.
• Milk productivity and cow characteristics had an impact on milk vitamin B_12_.
• Milk vitamin B_12_ was positively related to dietary fiber and negatively to energy.

## Graphical Abstract

**Figure:**
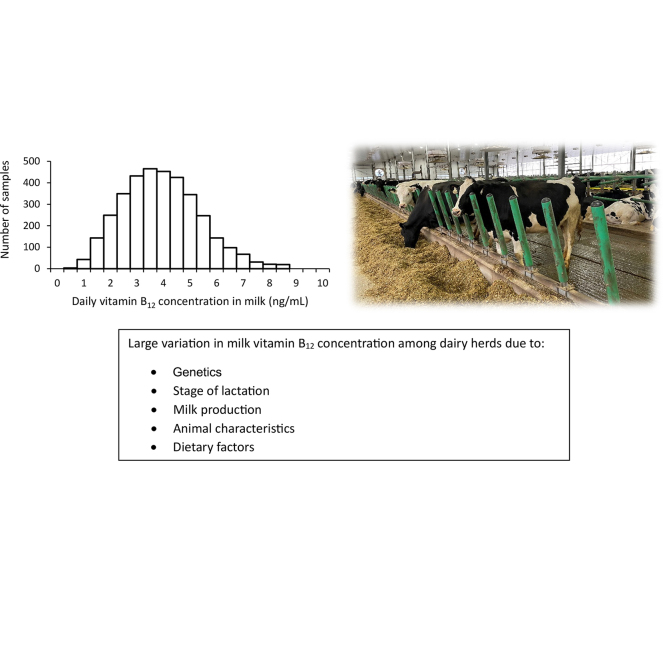
**Summary:** Milk is an excellent source of vitamin B_12_, but its concentration varies greatly among cows and herds. We intended to explain most of this variation by a multivariable statistical model using data from 3,533 Canadian Holstein cows from 99 herds. Our final model explained 79% of the vitamin B_12_ variation in milk and included factors related to genetic variation, milk productivity (negatively with milk yield and positively with milk fat and protein concentrations, following a similar lactation curve with a nadir at 50-55 days in milk), animal characteristics (positively with body weight and age at calving), and the diet (positively related to fiber and negatively to energy). Our results also show that milk vitamin B12 can be modified by genetic selection with a heritability of 0.37. To our knowledge, this is the most comprehensive assessment of factors affecting milk vitamin B_12_ concentration.

Vitamin B_12_ (**VB_12_**) is essential for animals and humans. In humans, this vitamin is important for preventing neuropathy, megaloblastic anemia, and gastrointestinal problems (Combs and McClung, 2022). Vitamin B_12_ differs from other B vitamins as it is only synthesized by bacteria when cobalt is available (Martens et al., 2002). Hence, as the rumen contains a myriad of bacteria synthesizing VB_12_, ruminant products are an excellent source of this vitamin for humans. Indeed, cow milk is a good dependable and natural source of VB_12_ (Walther et al., 2022); according to the National Academy of Sciences (1998), a 250-mL glass of milk provides 46% of the recommended dietary allowance of VB_12_ for humans above 13 years old. A recent study has shown that milk VB_12_ concentration was highly variable among herds; a 250-mL glass of milk fulfilled from 28% to 61% of the recommended dietary allowance (Duplessis et al., 2019). Given this wide variability and the importance of VB_12_ for humans, some authors studied factors explaining its variation in milk. It has been previously observed that genetic effects (Rutten et al., 2013; Duplessis et al., 2016; Gebreyesus et al., 2021), milk productivity and cow characteristics (Duplessis et al., 2019), rumen microbiota (Franco-Lopez et al., 2020), cow breed (Anthony et al., 1951; Duplessis et al., 2018), cow nutrition (Duplessis et al., 2016, 2019), rumen fermentation parameters (Duplessis et al., 2021), and seasons (Scott et al., 1984; Duplessis et al., 2016) have an effect on milk VB_12_. This shows that the regulation of milk VB_12_ concentration is multifactorial.

Increasing the knowledge on factors affecting milk VB_12_ variation is the first step to optimize and stabilize its concentration, hence strengthening the perception that milk is an excellent source of VB_12_ for consumers. This is particularly important as milk consumption has constantly declined over the last decades (Stewart et al., 2021). Thus, the primary objective of this study was to build a model that integrates several factors (genetic and nongenetic) affecting milk VB_12_ from a large dataset of Canadian Holstein cows. Another rationale was to evaluate the heritability (h^2^; i.e., the proportion of variation caused by genetic variation) of milk VB_12_ of Holstein dairy cows from a large dataset containing animals with a broad range of parities and DIM located in several herds.

All procedures were approved by the Animal Care Committee from Université Laval, QC, Canada, following the guidelines of the Canadian Council on Animal Care (2009). Animals and data collection were previously described by Duplessis et al. (2019). Briefly, 100 commercial dairy herds located in Québec, Canada, were involved in this cross-sectional study. To participate, it was required that Holstein was the main breed of the herd, that performance data were recorded through a DHI agency, and that cows were milked twice daily. Herds that fulfilled these requirements were contacted by phone and participation was on a voluntary basis. Cows were housed in tiestalls (n = 98) or freestalls (n = 2) and herd size ranged from 16 to 113 lactating Holstein cows. Three different feeding systems were used: (1) TMR (n = 31); (2) automatic component feeding (**ACF**; n = 49); and (3) manual component feeding (**MCF**; n = 20).

Herds were visited during 3 consecutive milkings (i.e., morning and evening milkings the first day and morning milking the second day of the visit). Each ingredient fed to the cows was sampled during the first morning milking visit and stored at −20°C until further analysis. Daily quantity of each ingredient given was recorded as previously reported (Duplessis et al., 2019). During evening milking of the first day and morning milking of the second day, milk yield was recorded, and milk samples were separately collected for each cow using calibrated in-line milk meters throughout the milking. A total of 4,340 Holstein cows (1,484 first, 1,093 second, and 1,763 third and more lactations) from 3 to 592 DIM were milk sampled. One aliquot of milk per milking was preserved with bronopol for major component analyses, and another one, without bronopol preservative, was kept at −20°C until VB_12_ analysis. Characteristics of cows (i.e., parity and DIM) and registration numbers were obtained from Lactanet (Canadian Network for Dairy Excellence, Sainte-Anne-de-Bellevue, QC, Canada). Body weight was estimated using heart girth circumference measurement (Yan et al., 2009).

Milk samples with bronopol from evening and morning milkings were immediately sent to the Lactanet laboratory for milk fat, protein, and lactose concentration analyses by mid-infrared spectroscopy using MilkoScan FT 6000 (Foss, Hillerød, Denmark). Milk VB_12_ of evening and morning milkings was analyzed in duplicate by radioassay using a commercial kit (SimulTrac B_12_/Folate-S; MP Biomedicals, Solon, OH; Duplessis et al., 2015). The interassay CV was 3.1%. Daily milk VB_12_ was calculated as follows: (evening milk VB_12_ concentration × evening milk yield + morning milk VB_12_ concentration × morning milk yield)/daily milk yield. Vitamin B_12_ was obtained in picograms per milliliter and transformed into nanograms per milliliter to facilitate numerical stability.

Feed samples were dried at 55°C for 48 h, ground at 1 mm, and sent at SGS Canada (Guelph, ON, Canada) for nutrient concentrations based on AOAC International (2005) as previously described (Fadul-Pacheco et al., 2017). Percentage of inclusion of each ingredient in the diet was computed on a DM basis. Nutrient composition of the diet was then calculated by multiplying the inclusion percentage of each ingredient on a DM basis by its respective nutrient composition.

After merging the Lactanet registration number file of the 100 dairy herds with the sample collection dataset file based on cow identification numbers, there were 4,672 records in the dataset. Data edition was then needed to remove irrelevant records. After the edition for duplicate, mismatched, or invalid registration numbers, 4,407 records remained. When merging with the daily milk VB_12_ file, 278 records were excluded due to missing milk VB_12_ data. Only animals with age at calving between 21 and 96 mo were kept (158 records excluded). There were insufficient numbers of animals in parities 7 and greater to warrant retaining them and they were dropped from the analyses (n = 89). Records with no milk production information (n = 27) as well as milk VB_12_ greater than 9.0 ng/mL were deleted (n = 29) because they were considered outliers or coming from unhealthy cows (Duplessis et al., 2018). Records were dropped because animal identification was inconsistent from the registration number file of Lactanet and the daily milk VB_12_ file (n = 90). From the latter, one herd was dropped because all records were inconsistent (n = 34). All records from cows that were not pure Holstein or one of their parents were not pure Holstein were removed from the dataset (n = 127). Finally, records of cows with more than 430 DIM were deleted (n = 76). The final dataset had 3,533 records (1,239 first, 932 second, and 1,362 third and more lactations), one record per cow from 99 herds. From these, pedigrees (ancestors) were traced back for 3 complete generations from each of their sires and dams using the Holstein breed pedigree files maintained by Lactanet. This produced a total of 12,980 animal identities. Noninformative animals were pruned out (i.e., animals who had both parents unknown, no actual data records themselves, and only one progeny). There remained 10,021 identities for use in the subsequent genetic analysis.

Descriptive statistics were obtained using Proc UNIVARIATE of SAS (version 9.4; SAS Institute, 2012). The current assessment uses a subset of cows from the study of Duplessis et al. (2019) in which the authors concluded that milk production, diet, and cow characteristics explained 52% of the milk VB_12_ variation. However, they did not include genetic relationships among animals in their model, and more exclusion criteria as described above were applied in the current work leading to a suppression of 807 cows relative to the dataset of Duplessis et al. (2019). These authors used principal component (**PC**) analysis to overcome multicollinearity among independent variables related to dietary composition and ingredients. Then, they used PC as independent variables in a multivariable model aiming to study the relationship between daily milk VB_12_ as a dependent variable. Thus, a similar approach as Duplessis et al. (2019) with slightly different independent variables was used in the current article to also include the impact of genetic variation on VB_12_ in the model for a better integration. Briefly, 26 independent continuous variables pertaining to diet composition (NE_L_ [Mcal/kg of DM], CP, RUP, starch, ADF, NDF, NDF from forage, lignin, NFC, fat [% of DM], and Co [mg/kg of DM]), diet ingredients (corn silage, chopped or baled mixed grass-legume silage, hay, concentrate, corn, other cereals [i.e., oat, barley and wheat], soy products, commercial protein supplement, commercial energy supplement, and fat supplement [% of DM]), and milk production (kg/d) and composition (%; fat, protein, and lactose concentrations) were used in the PC analysis using Proc FACTOR of SAS. To be retained for further analysis, a PC should explain at least 5% of the total variance. A VARIMAX rotation was applied to the PC to facilitate their interpretation. To be part of a PC, a variable should have the highest coefficient among the obtained PC. Then, each PC can be considered as an independent variable in a multiple regression analysis without multicollinearity among the PC. From the pedigree (3 generations back on both the sire and dam pathways of each sampled cow), inbreeding values, which are associated with a loss of genetic diversity (Meuwissen et al., 2020), were calculated according to Quaas (1976).

The model used in WOMBAT (Meyer, 2007) to estimate genetic parameters in addition to the PC and other independent factors was

*Y_ijklmpstuw_* = *µ* + *FS_i_* + *month_j_* + *herd_ijk_* + *DIM_l_* + e^(−0.05×DIM)^*_l_* + *parity_m_* + *AaC_mp_* + *inbreeding_s_* + *BW_t_* + *PC_u_* + *animal_ijklmpstuw_* + *e_ijklmpstuw_*,

where *Y_ijklmpstuw_* is the dependent variable, that is, daily VB_12_ in milk collected from the *w*th cow from the *i*th feeding system (FS) in the *j*th month of sampling, *k*th herd nested within the *i*th FS in the *j*th month, which cow was sampled when she was at her *l*th DIM at *m*th parity and calved at the *p*th age at calving (*AaC*) in months nested within parity, with inbreeding level *s* with *t*th BW and *u*th PC; *µ* is the overall mean; *FS_i_* is the fixed effect of FS (TMR, ACF, and MCF); *month* is the fixed effect of sampling month; *herd_ijk_* is the random effect of the *k*th herd nested within the *i*th FS and the *j*th month of sampling [*k* = 1 to 99; *herd_ijk_* ∼*N*(0, *σ*^2^)]; *DIM_l_* is the fixed effect of DIM of the cow when the sample was collected (*l* = 1 to 430); e^(−0.05×DIM)^*_l_* is to take into account the Wilmink curve model (Wilmink, 1987); *parity_m_* is the fixed effect of parity of the sampled cows (*m* = 1 to 6); *AaC_mp_* is the fixed effect of *AaC* (*p* = 21 to 96 mo) nested within parity; *inbreeding_s_* is the fixed effect of the inbreeding level category (increment of 1%; *s* = 1 to 15%); *BW_t_* is the fixed effect of estimated cow BW; *PC_u_* is the fixed effect of PC (*u* = 1 to 6); *animal_ijklmpstuw_* is the random effect of the cow to include animal and genetic relationships ∼*N*(0, **A***σ*^2^), where **A** is the numerator relationship matrix; and *e_ijklmpstuw_* is the residual error term. Proc REG of SAS was used to compute pseudo-R^2^ from predicted milk VB_12_ from the final model and observed values. The predicted values were obtained from WOMBAT. The residuals were visually assessed for normality and homoscedasticity. Results were considered significant when *P* ≤ 0.05. Heritability was computed as per Poulsen et al. (2015) and Gebreyesus et al. (2021), including or not the herd variance.

On average, cows in the current assessment yielded 32.4 kg/d of milk (Table 1). Milk VB_12_ ranged from 0.68 to 8.96 ng/mL and averaged 4.18 ± 1.45 ng/mL (Table 1). A typical ration had a forage:concentrate ratio averaging 67:33 with 15.2% of CP, 1.57 Mcal/kg of NE_L_, 21.8% of ADF, 37.4% of NDF, 40.7% of NFC, and 0.60 mg/kg Co on a DM basis. Among the 10,021 identities used for subsequent genetic analyses, inbreeding values ranged between 0 and 15%; 83% of the animals had an inbreeding value between 0% and 1%, 10% between 1% and 2%, and the remaining 7% of animals had inbreeding coefficients above 2%. Current inbreeding values were lower than reported for Canadian Holstein cattle born from 1980 to 2004 (3.20%) (Sewalem et al., 2006).

**Table 1:** Descriptive statistics regarding the 3,533 Holstein dairy cows located in 99 herds included in the analysis

Item	Average	SD	Minimum	Maximum
Daily milk yield (kg)	32.4	9.2	7.1	66.9
Daily milk fat concentration (%)	4.10	0.58	1.99	6.66
Daily milk protein concentration (%)	3.34	0.34	2.40	4.62
Daily milk lactose concentration (%)	4.58	0.18	3.70	5.15
Daily milk vitamin B_12_ concentration^1^ (ng/mL)	4.18	1.45	0.68	8.96
Parity	2.3	1.3	1	6
DIM	172	101	3	429
Estimated BW^2^ (kg)	677	56	516	892

1 Obtained from evening and morning milk vitamin B_12_ concentration samples weighted according to respective milk yield.

2 Estimated using heart girth circumference according to the equation of Yan et al. (2009).

Six PC fulfilled the requirement of explaining at least 5% of the total variance. These 6 PC were then retained for further analysis and altogether explained 59% of the total variance; 22%, 11%, and 8% of the variance were explained by PC1, PC2, and PC3, respectively. The first PC was negatively associated with dietary fiber (NDF from forage, NDF, ADF, and lignin) and positively with indicators of dietary energy (NFC, starch, NE_L_, and percentage of concentrate). Lactation performance was grouped into the PC2 (negatively with milk yield and positively with milk fat and protein concentrations). The third PC was positively related to dietary protein (CP and RUP) and Co concentrations. The fourth PC was negatively associated with mixed silage as bale, whereas it was positively associated with chopped mixed silage stored in silo. Dietary fat and percentages of commercial fat inclusion were positively associated, whereas percentage of corn silage was negatively related to PC5. The last PC, PC6, had a positive relationship with percentage of soy products.

The novelty of the current assessment is to allow integrating the impact of genetic variation, animal characteristics and milk productivity, and diets on milk VB_12_ in the same multivariable statistical model. It is noteworthy that the independent variables that affected VB_12_ in milk are similar to results reported by Duplessis et al. (2019), the current assessment using a subset of their cows and a comparable methodology. Nevertheless, the random effect of the cow to include animal and genetic relationships and the fixed effect of inbreeding were not previously added.

Table 2 depicts which independent variables had a significant relationship with daily milk VB_12_. Milk VB_12_ differed according to DIM and the exponential term of DIM (*P* < 0.001; i.e., the effect of DIM was well-modeled with the Wilmink regression). Age at calving and estimated BW were positively related to VB_12_ in milk (*P* < 0.001). Out of 6 PC, 2 were retained in the final model because they reached significance. The one pertaining to dietary fiber and dietary indicators of energy (PC1) was negatively associated and the one related to lactation performance (PC2) was positively related to milk VB_12_ (*P* < 0.001). Results indicate that milk VB_12_ increased as dietary ADF, NDF, NDF from forages, and lignin increased, whereas an inverse relationship was obtained for dietary NE_L_, starch, NFC, and concentrate percentages as similarly observed by Duplessis et al. (2016, 2019). van den Oever et al. (2021) observed that milk VB_12_ was greater when cows ingested silage instead of hay as the sole forage. Our results did not support this finding, possibly because all herds fed their cows with a blend of 2 or more different forages in contrast to the study of van den Oever et al. (2021). The relationship with PC2 showed that milk yield was negatively and milk fat and protein concentrations were positively related to milk VB_12_. Hence, cows producing low milk yield with high milk fat and protein concentrations will have greater milk VB_12_ than the opposite. Results on the relationship between DIM, exponential term of DIM, milk performance, and milk VB_12_ are similar to the ones reported in Duplessis et al. (2019), in which the lactation curve of VB_12_ reached the nadir at 50 to 55 DIM. Results suggest that as AaC increased, milk VB_12_ also increased. There was no significant effect of parity over and above the effect of age at calving (*P* = 0.43); parity was therefore dropped and age at calving was kept in the model. It is noteworthy that age at calving is closely related to parity. Similarly, in the model used by Duplessis et al. (2019), milk VB_12_ increased as parity increased.

**Table 2:** Parameter estimates included in the multivariable model and heritability of daily milk vitamin B_12_ concentration (ng/mL) of 3,533 Canadian Holstein cows located in 99 herds

Parameter	Estimate	SEM	*P*-value
Multivariable model			
DIM	0.002851	0.0002034	<0.001
e^−0.05×DIM^	0.6752	0.1634	<0.001
Age at calving (mo)	0.006693	0.001344	<0.001
Estimated cow BW (kg)	0.002107	0.0004021	<0.001
PC1^1^	−0.0932	0.0340	<0.001
PC2^2^	0.5303	0.0262	<0.001
Heritability (h^2^)			
Phenotypic variance (× 10^6^)^3^	1.55	0.07	—
h^2^asσa2/σa2(σa2+σherd2+σe2)(σa2+σherd2+σe2)^4^, ^5^	0.27	0.04	—
σherd2/σherd2(σa2+σherd2+σe2)(σa2+σherd2+σe2)^5^	0.25	0.03	—
h^2^[asσa2/σa2(σa2+σe2)(σa2+σe2)]^5^, ^6^	0.37	0.05	—

1 PC1 = principal component 1, negatively associated with dietary fiber (NDF from forage, NDF, ADF, and lignin) and positively with indicators of dietary energy (NFC, starch, NE_L_, and percentage of concentrate).

2 PC2 = principal component 2, negatively associated with milk yield and positively with milk fat and protein concentrations.

3 Sum of the 3 variance components: genetic variation of the animal, herd variation, and residual variation.

4 According to the calculation of Poulsen et al. (2015).

5 Variance components: $\sigma_{a}^{2}$ = genetic variation of the animal (0.421 ± 0.065); $\sigma_{herd}^{2}$ = herd variation (0.384 ± 0.063); $\sigma_{e}^{2}$ = residual variation (0.739 ± 0.053).

6 According to the calculation of Gebreyesus et al. (2021).

The h^2^ was 0.27 ± 0.04 when considering the herd variance and 0.37 ± 0.05 without the herd variance (Table 2). To our knowledge, the current report is the most comprehensive assessment on genetic variation in milk VB_12_, including more than 3,500 Canadian Holstein cows at all lactation stages and parities. The first report demonstrating a genetic variation among animals for milk VB_12_ used 544 primiparous Dutch Holstein cows between 76 and 282 DIM (Rutten et al., 2013). Other previous genetic evaluations of VB_12_ in milk included 343 early-lactating Canadian Holstein (Duplessis et al., 2016) and 341 Danish Holstein cows (Gebreyesus et al., 2021). In the literature, 2 different h^2^ calculations have been found (Rutten et al., 2013; Poulsen et al., 2015), considering or not the herd variance. We obtained a greater value when the herd variance was not considered as per Poulsen et al. (2015). Our results are in agreement with previous experiments reporting an h^2^ of 0.37 when disregarding the herd variance in the calculation (Rutten et al., 2013; Gebreyesus et al., 2021). Moreover, Duplessis et al. (2016) estimated the h^2^ value to 0.23 when the herd variance was included in the calculation, similar to the 0.27 of the current study. These studies showing moderate h^2^ for milk VB_12_ indicate that there is a significant additive genetic variability, which could be exploited to increase milk VB_12_ supplied to the consumer. Our results reported for the first time that inbreeding did not have an impact on milk VB_12_, although current inbreeding values were lower than previously reported in Canadian Holstein.

The final WOMBAT model including significant independent variables with the random effect of the cow to include animal and genetic relationships explained 79% of the milk VB_12_ variability (pseudo-R^2^ of 79%). Not including the animal and genetic relationships gave a pseudo-R^2^ of 43% (i.e., animal and genetic explained about 36% of the variation) and further excluding DIM and e^(−0.05×DIM)^ gave a pseudo-R^2^ of 27% (i.e., DIM and e^(−0.05×DIM)^ explained about 16% of the variation). Interestingly, PC2, which was the third largest source of the variation, only contributed to 6% of the variation, and PC1, 1% of the variation. Duplessis et al. (2019) concluded that 52% of the milk VB_12_ variability was explained by milk production, cow characteristics, and diet composition, indicating a substantial improvement by including the animal and genetic relationships.

In summary, these results highlight that several factors affect milk VB_12_. With a h^2^ of 0.37 when not considering the herd variance, this strengthens the idea that VB_12_ in milk can be increased by genetic selection. The impact of other factors such as ruminal microbiome and breed of the cow on VB_12_ can be further explored in an integrated model from data collected in several herds.
